# Inertial Sensor-Based Touch and Shake Metaphor for Expressive Control of 3D Virtual Avatars

**DOI:** 10.3390/s150614435

**Published:** 2015-06-18

**Authors:** Shashidhar Patil, Harinadha Reddy Chintalapalli, Dubeom Kim, Youngho Chai

**Affiliations:** Graduate School of Advanced Imaging Science, Multimedia and Film Chung-Ang University, Seoul 156-756, Korea; E-Mails: patil.shashidhar@hotmail.com (S.P.); harinath@cau.ac.kr (H.R.C.); rodin1818@hotmail.com (D.K.)

**Keywords:** inertial sensors, gestural interfaces, expressive control, gesture recognition, gesture variations, interactive systems, touch and shake, virtual avatar

## Abstract

In this paper, we present an inertial sensor-based touch and shake metaphor for expressive control of a 3D virtual avatar in a virtual environment. An intuitive six degrees-of-freedom wireless inertial motion sensor is used as a gesture and motion control input device with a sensor fusion algorithm. The algorithm enables user hand motions to be tracked in 3D space via magnetic, angular rate, and gravity sensors. A quaternion-based complementary filter is implemented to reduce noise and drift. An algorithm based on dynamic time-warping is developed for efficient recognition of dynamic hand gestures with real-time automatic hand gesture segmentation. Our approach enables the recognition of gestures and estimates gesture variations for continuous interaction. We demonstrate the gesture expressivity using an interactive flexible gesture mapping interface for authoring and controlling a 3D virtual avatar and its motion by tracking user dynamic hand gestures. This synthesizes stylistic variations in a 3D virtual avatar, producing motions that are not present in the motion database using hand gesture sequences from a single inertial motion sensor.

## 1. Introduction

Rapid developments in computing technology and recent advances in user interfaces have replaced the conventional interaction tools such as keyboard and mouse. In daily life, the use of human gestures to communicate and control interactive applications such as computer games and humanoid interfaces in virtual environments is increasing. Gestural interfaces enable users to naturally interact with the virtual environment. Designing a dynamic gesture interface system to recognize a user’s actions and corresponding reaction from the application in real time is a challenging task. Gestures are meaningful body motions, and can include static or dynamic physical movements of the fingers, hands, face, or body for interaction with the environment.

Gestural interfaces are currently being developed for applications such as virtual reality, sign language, and remote control using different motion tracking and gesture recognition techniques [1]. The use of different imaging and tracking devices are required to recognize the gestures. A user interface based on the conventional keyboard and mouse is not suitable for interactive and dynamic environments; rather, devices that sense a user’s body must be used. Many researchers have developed gesture recognition techniques using vision-based methods [2].

In [3], the authors present a motion-capture-based performance animation system that maps detailed user motion to a virtual avatar; an optical motion capture system is described in [4]. However, motion-capture-based methods require significant user set-up time. They are limited by the capture environment and lighting conditions, and their cost makes them impractical for personal use. Gesture recognition can be used in entertainment and serious games to control avatars or interact with virtual worlds. Low-cost motion-capture depth sensor devices, such as Microsoft’s Kinect, are widely used for body movement recognition [5]. In [6], a Kinect was used to capture depth images for American Sign Language (ASL) detection. In [7], both depth and color information from a Kinect were used to detect hand and gesture recognitions. The Leap Motion Controller [8] hand-motion sensing device also employs a depth camera to track user hand movements in a constrained environment. Moreover, vision-based sensors suffer from occlusion problems. These vision-based techniques are limited to wearability and have prohibitive computational costs, are sensitive to lighting conditions, and require a large workspace.

Inertial-sensor-based gesture recognition has been successfully used in mobile and pervasive computing [9,10,11,12]. Interaction devices such as the Nintendo Wii [13] and Sony Motion Controller have been widely used in computer games, and allow interaction with the game by employing a user’s natural motions, such as moving the device, hand, or full body. This approach is appealing because it is cost-effective and low power, and can easily provide intuitive expressions through the linear and angular acceleration generated by hand motions.

In [14], the authors describe the use of accelerometers to search for matched motion from a motion database. In [15], a small number of inertial motion sensors are used as a performance animation interface that tracks human motion to identify identical motion from a database and reconstruct character animation. These approaches focus on finding an example of the input motion in a database. The fusion of microelectromechanical systems (MEMS)-based inertial sensors and low-resolution vision sensors has been used for 2D human gesture tracking and recognition using an extended Kalman filter [16].

Existing gesture interface techniques are cumbersome and lack control over the expressive movements of user motion. Gesture data are collected through observations of the human body using sensors; the received sensor data are used to train the model for different activities. These trained models are subsequently used to predict the activities of new gestures. Such interface systems lack dynamic control, and are ineffective in mapping the user intentions. Therefore, interactive applications such as games and virtual worlds would benefit greatly from dynamic control and adaptive methods.

Recognition of user gestures is important in gesture-based interaction techniques. Many gesture recognition methods exist for 3D spatial gestures, such as hidden Markov models (HMMs), support vector machines (SVMs), and dynamic-time-warping (DTW). In [17,18], HMM-based approaches were shown to be effective at increasing the recognition rate of inertial sensing-based gesture recognition. However, HMM classifiers are expensive on account of their computational complexity; moreover, they require more than one training sample to efficiently train the model and obtain better recognition rates.

The DTW algorithm measures similarity and computes the distance between two signals that may vary in terms of time or speed. DTW is effective even when only one training dataset is available. Furthermore, it is easy to execute, computationally efficient, and more accurate for time series data than other statistical gesture recognition methods. Many researchers have demonstrated the effectiveness of the DTW algorithm [19,20,21]. For example, [22] applied DTW for the recognition of musical gestures, and [23] employed DTW for personalized gesture recognition. Trajectory-based gesture recognition for Arabic numerals has also applied DTW [16].

An expressive control interface system that enables users to author and control a 3D virtual avatar and its motion is needed. The present research is motivated by this need for an intelligent, dynamic, and user-intuitive gesture interface system with expressive control that uses gesture variation for continuous interaction. Our goal is to provide an interactive control interface that will enable users to expressively author and control the 3D virtual avatar motion in real time, allowing users intended motion features to be perceived.

We herein present an interactive gesture-based control interface for authoring and controlling a 3D virtual avatar and its motion by tracking user dynamic hand gestures with a single six-degrees-of-freedom (6DOF) wireless inertial motion sensor. It is not easy to author and control a high-dimensional 3D virtual avatar using a single inertial motion sensor. To overcome the dimensionality problem and the lack of sensor inputs, we consider data-driven motion synthesis using a small human motion database to author a high-dimensional virtual avatar. In this paper, we show that the stylistic variations of 3D avatar motions can be quickly and easily generated from a single example of motion data and user-specified dynamic gestures using a single inertial motion sensor.

Statistical methods for controlling character animation have been used by many researchers. For instance, a method that uses a dynamic Bayesian network to model spatiotemporal variations of human motion has been developed [24,25]. The authors of [26] used a statistical dynamic model of motion capture data to generate animation. The above methods are heavily dependent on the motion modeling topology, initialization, and latent variation parameters. To handle stylistic variations, these latent variable models must be manually adapted. In [27], principal component analysis (PCA) is used to decompose sets of motion data; PCA coefficients are then used to synthesize new motions with different styles.

We adopt a data-driven motion synthesis method. Specifically, a statistical approach that uses independent component analysis (ICA) is employed as a motion decomposition method for the analysis and synthesis of stylistic avatar motion. The idea of using ICA to extract meaningful motion features from motion data was proposed by [28]. In [29], styles were separated from motion data using ICA to decompose a single motion into linear combinations of independent components.

Our approach uses a distinct method of generating stylistic motions from a single motion example. The proposed expressive control of a 3D virtual avatar employs a dynamic gesture interface using a DTW-based recognition technique to control avatar motion with user hand gestures. This technique generates various styles of avatar motion with spatial and temporal variations from a single motion example by decomposing the avatar motion data and dynamic gesture data into linear combinations of independent components.

The remainder of this paper is organized as follows: Section 2 provides an overview of our system interface. The inertial motion sensor is presented in Section 3. In Section 4, the implementation of an appropriate dynamic gestural interface is explained. Gesture-to-motion mapping with expressive motion synthesis is described in Section 5, and experimental results are discussed in Section 6. Section 7 concludes our paper with suggestions for future work.

## 2. System Overview

**Figure 1:**
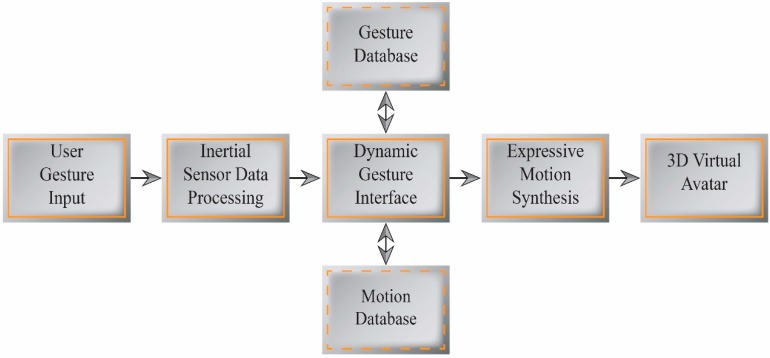
Dynamic gesture-based expressive control interface system.

Our system uses a single inertial motion sensor with a dynamic gesture-based interactive control interface to author and control avatar actions according to user intentions. From user-generated gesture data, a DTW-based dynamic gesture mapping interface extracts the significant motion components from example motion data, and an expressive motion synthesis module modifies these motion components with the extracted features and components from the user hand gesture. This enables the synthesis of new stylistic motion and variations that effectively convey the user intentions in real time. Figure 1 illustrates the overall process of our dynamic gesture-based expressive control interface system.

## 3. Inertial Motion Sensor

### 3.1. Wireless Motion Controller

To enable effective 3D motion tracking, we employ commercial solid-state MEMS inertial sensors in a custom-made wireless motion sensor system using tri-axis magnetic, angular rate, and gravity (MARG) measurements. A sensor fusion algorithm then enables the user’s hand to be tracked with 6DOF in 3D space. It is well known that inertial sensor measurements contain a significant amount of noise. If the measurements are not carefully obtained, this can result in inaccurate sensor fusion output. We programmed our wireless motion sensor system with sophisticated automatic calibration algorithms and a quaternion complementary filter sensor fusion algorithm [30]. This filter computes quaternions relative to the direction of gravity and the Earth’s magnetic field.

In this section, we use a notation system of leading superscripts and subscripts, similar to the system used in [31], to denote the relative frame of orientations and vectors. For example, qtbe describes the quaternion of sensor body frame b relative to Earth frame e at time t, and Aestb is an estimated vector described in frame b. The ⊗ operator denotes a quaternion product.

**Figure 2:**
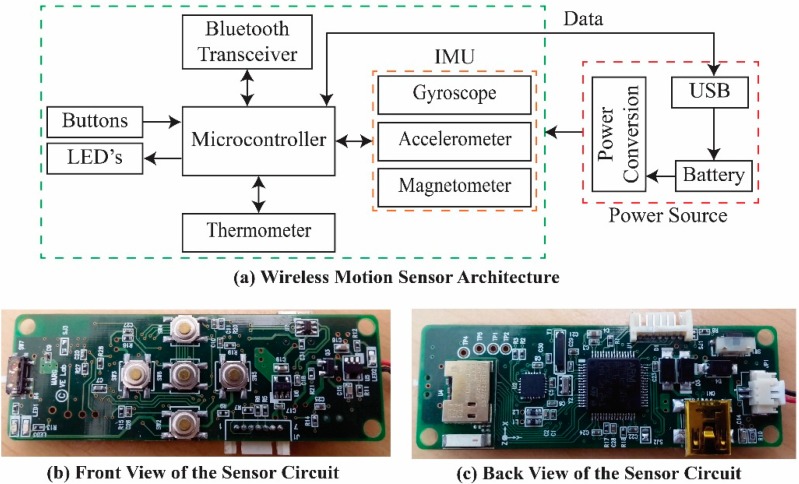
Wireless motion sensor system.

The wireless motion sensor system shown in Figure 2 incorporates a tri-axis gyroscope, tri-axis accelerometer, and tri-axis magnetometer (9-axis MEMS sensor MPU-9150, InvenSense, San Jose, CA, USA). These have selectable ranges of up to ±2000º/s, ±8 g, and ±1200 μT, respectively. The gyroscope measures angular rates $\omega_{x}$, $\omega_{y}$, and $\omega_{z}$ about the *x*, *y*, and *z* axes of the sensor body frame; the accelerometer measures accelerations $a_{x}$, $a_{y}$, and $a_{z}$; and the magnetometer measures magnetic fields $m_{x}$, $m_{y}$, and $m_{z}$.

A digital thermometer is included to compensate for time-varying temperature biases in the MEMS sensors. The sensors, along with buttons, status LEDs, and a Bluetooth transceiver, are connected to a microcontroller for collecting, controlling, and processing data. The motion data are acquired and processed at 100 Hz.

### 3.2. Quaternion Complementary Filter

**Figure 3:**
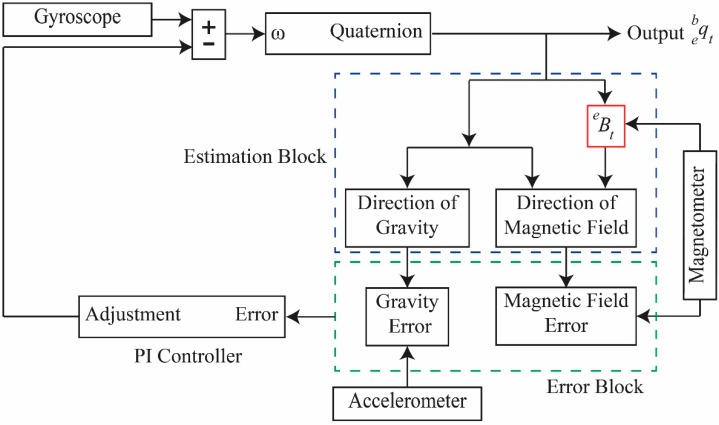
Complementary filter block diagram.

Sensor fusion attitude and heading reference system (AHRS) algorithms compute the 3D attitude from a gyroscope. These algorithms use additional reference measurements from an accelerometer and a magnetometer to compensate for drift. A complementary filter uses the gyroscope data to compute the quaternion rotation. To compensate for the drift in the quaternion rotation, a gravity vector of the accelerometer is used along the *x* and *y* axes; a heading vector of the magnetometer is used for *z*-axis drift. Additionally, a complementary filter incorporates a magnetic distortion compensation technique to correct for nearby sources, such as metal structures or power supply busses. This filter has a proportional and integral (PI) controller with two tuning parameters (proportional gain $K_{p}$ and integral gain $K_{i}$), which are used to tune the algorithm. A block diagram of this filter is shown in Figure 3.

#### 3.2.1. Quaternion Computation

If the angular rates in radians per second are arranged into a vector, as shown in Equation (1), then the quaternion derivative describing the rate of change of the Earth frame relative to the sensor body frame at time *t* can be calculated as shown in Equation (2) [32]. Equation (3) describes how to compute the attitude quaternion at time *t* by numerically integrating the expression in Equation (2). In Equation (3), *Δt* is the sampling period, qt−1eb is the previous attitude quaternion, and $q_{1},\;q_{2},\;q_{3},$ and $q_{4}$ represent elements of the quaternion:

(1) ωtb=[0 ωx ωy ωz]

(2) qt*eb=12qt−1eb⊗ωtb

(3) qteb=[q1 q2 q3 q4]=qt−1eb+qt*ebΔt

#### 3.2.2. Estimation Block

The normalized magnetic field measurements can be arranged into a vector, as shown in Equation (4). This vector is incorporated into the estimation block, along with the previous attitude quaternion qt−1eb, to estimate the direction of gravity and the magnetic field. Equations (5) and (6) are used to compensate for magnetic distortions; they are represented by a red box in the block diagram, and qt−1*eb represents the conjugate of the quaternion at time t−1. Equations (7) and (8) estimate the direction of the gravity and magnetic field vectors, respectively:

(4) Mtb=[0 mx my mz]

(5) Hte=[0 hx hy hz]=qt−1eb⊗Mtb⊗qt−1*eb

(6) Bte=[0 bx by bz]=[0  hx2+hy2   0  hz]

(7) Gest,tb=[gxgygz]=[2(q2q4−q1q3)2(q1q2+q3q4)q12−q22−q32+q42]

(8) Mest,tb=[wxwywz]=[2bx(0.5−q32−q42)+2bz(q2q4−q1q3)2bx(q2q3−q1q4)+2bz(q1q2+q3q4)2bx(q1q3+q2q4)+2bz(0.5−q22−q32)]

#### 3.2.3. Error Block

The error block computes the total error, which is the sum of gravity and the magnetic field error vectors, by incorporating the normalized acceleration and magnetic field measurements along with the results of Equations (7) and (8). Further inspection of Equation (9) reveals that the error is the cross-product between the estimated and measured quantities of gravity and the magnetic field:

(9) $$E=\left[\begin{array}{c} e_{x}\\ e_{y}\\ e_{z} \end{array}\right]=\left[\begin{array}{c} \left(a_{y}g_{z}-a_{z}g_{y}\right)+(m_{y}w_{z}-m_{z}w_{y})\\ \left(a_{z}g_{x}-a_{x}g_{z}\right)+(m_{z}w_{x}-m_{x}w_{z})\\ \left(a_{x}g_{y}-a_{y}g_{x}\right)+(m_{x}w_{y}-m_{y}w_{x}) \end{array}\right]$$

#### 3.2.4. Proportional Integral Controller

The PI controller in the block diagram is the heart of the algorithm; it provides two tuning parameters, proportional gain $K_{p}$ and integral gain $K_{i}$. $K_{p}$ determines how quickly the algorithm output converges to the accelerometer and magnetometer measurements. In other words, $K_{p}$ enables us to tune the degree to which the sensors are trusted; a low value denotes greater trust of the gyroscope; e.g., $K_{p}=0$ indicates that the accelerometer and magnetometer data will be ignored; $K_{p}=0.5$ is suitable in most cases. $K_{i}$ corrects for gyroscope bias; we assume $K_{i}=0$, because calibrated data is supplied to the filter. Equation (10) outputs the adjustment that will be sent to the correction block. The angular velocity at time t, $\omega_{t}$, is corrected using this adjustment:

(10) $$U_{t}=\left[\begin{array}{c} K_{p}e_{x}+K_{i}j_{x}\\ K_{p}e_{y}+K_{i}j_{y}\\ K_{p}e_{z}+K_{i}j_{z} \end{array}\right]\;where\;J_{t}=\left[\begin{array}{c} j_{x}\\ j_{y}\\ j_{z} \end{array}\right]=\{\begin{array}{c} J_{t-1}+E*\Delta t\;\;\;\;\;\;K_{i}>0\\ 0\;\;\;\;\;\;\;\;\;\;\;\;\;\;\;\;\;\;\;\;\;\;otherwise \end{array}$$

## 4. Dynamic Gesture Interface

Real-time hand gesture recognition using an inertial sensor is a challenging task, because the gestures performed by different individuals can vary dramatically. We are especially interested in free-space hand motion gestures. A hand gesture involves symbolic and continuous data. Hand gesture motions are primarily used for natural and continuous interactions among people. These gestures reflect emotional states, and can be intentionally manipulated or constrained. The constrained, symbolic, and qualitative nature of hand gestures can be an advantage for a dynamic gesture interface system.

**Figure 4:**
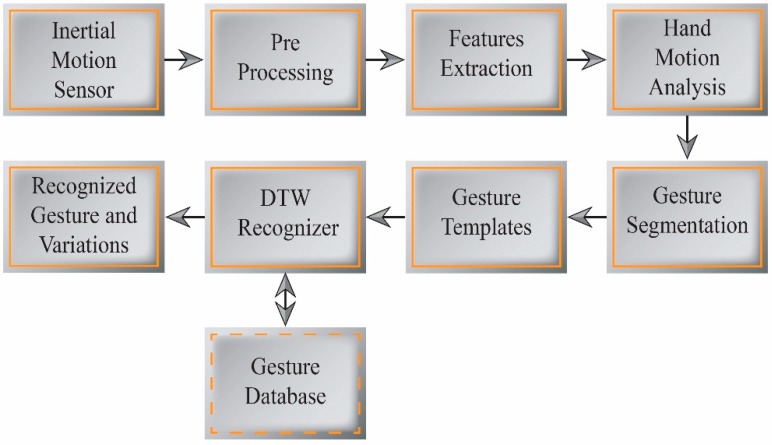
Hand gesture segmentation and recognition.

Existing gesture recognition techniques identify the gesture being performed from the motion sensor data. Our goal is to not only recognize the particular gesture being performed, but also to recognize how that gesture is being performed. The present approach assesses the gesture expressivity from variations in the gesture performance, and uses these variations to design an interactive avatar control application. We employ two techniques to develop expressive interaction, namely gesture tracking and gesture recognition. A DTW-based algorithm is used to estimate the gesture variations as well as to perform real-time gesture recognition. Figure 4 depicts a block diagram of our gesture segmentation and recognition process.

### 4.1. Hand Gesture Tracking and Segmentation

MARG sensor signals generated from a wireless motion sensor by hand movements are transmitted to a PC via a Bluetooth transceiver. The measured signals always contain noise and errors from both the sensor and involuntary user movements. To eliminate the noise and errors, we apply a preprocessing procedure to the acquired signals. This procedure uses signal calibration to reduce sensitivity and offset errors from the raw signals, and removes high-frequency noise from the calibrated signals via low-pass filtering.

The acceleration data contain motion-induced acceleration and gravity components. The gravity component is handled as noise, and is thus removed, because it does not depend on user motion. To compensate for gravity, we use the direction of gravity estimated from the quaternion complementary filter by Equation (7).

The quaternion $q_{n}$, which represents the orientation of the sensor frame, is used to transform the sensor-coordinated acceleration into an Earth coordinate using the quaternion operator given by Equation (11). After obtaining the acceleration from the Earth coordinate system, the gravitational acceleration G is subtracted from the acceleration to obtain motion-induced acceleration, as shown in Equation (12):

(11) $$a_{e}=q_{n}\left(t\right)⊗a_{s}⊗q_{n}^{*}(t)$$

(12) $$accel\left(t\right)=a_{e}\left(t\right)-G$$

Each gesture can be considered as an ordered sequence of segments. We employ a computational method to automatically segment an expressive gesture into a sequence of symbols. This simplifies the gesture recognition process. Segmentation enables the early recognition of gestures and the estimation of gesture variations with respect to a learned reference template. For our inertial sensor-based hand gestures, the accelerometer and gyroscope data from the motion sensors are processed and segmented for improved recognition efficiency. This also allows gesture variation features to be extracted from each gesture action in real time.

We employ user hand-motion constraints for gesture segmentation, as this is simple and effective for real-time use. The magnitude of linear acceleration and angular rate data from the user hand motions are calculated by Equations (13) and (14) for the segmentation of gesture actions into candidate gesture templates:

(13) $$AccelMag=\sqrt{accel_{x}^{2}+accel_{y}^{2}+accel_{z}^{2}}$$

(14) $$GyroMag=\sqrt{gyro_{x}^{2}+gyro_{y}^{2}+gyro_{z}^{2}}$$

Using a threshold-based detection method, we segment the performed gestures into candidate gesture templates. We use a small constant, such as 0.2 G for the acceleration threshold, to detect the occurrence of segmentation. In the evaluations, using only an acceleration threshold led to unexpected gesture segmentations; therefore, we employ a temporal threshold with the acceleration threshold. Segments that occur within the same temporal threshold are assumed to be the same segments, and are thus combined.

Similarly, for the gyro threshold, a small constant such as 20°/s determines whether segmentation has occurred. In our evaluations, gyro-based segmentation produced no unexpected effects. From this, we can conclude that gyro-based segmentation is more accurate than acceleration-based segmentation.

**Figure 5:**
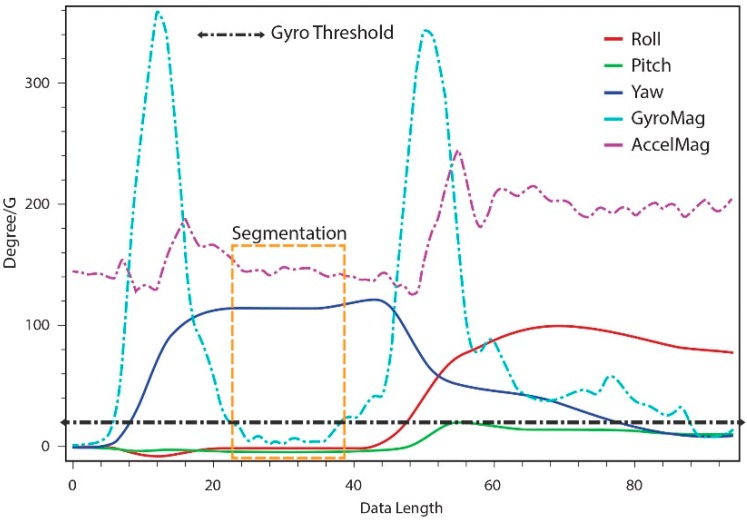
Acceleration and gyro-based gesture segmentation.

Our gesture segmentation process uses both acceleration and gyro-based calculations; the high-accuracy gyro-based segmentation validates the gesture segments made using acceleration-based detection, as shown in Figure 5. The segments simplify the process of gesture classification, and the real-time use of hand motion constraints for segmentation reduces the unwanted segmentation of gesture data compared to sliding window and other approaches for the online segmentation of time series data.

The quaternion output from the motion sensor is transformed into equivalent Euler sequences of rotation angles. This output is used as gesture feature parameters, which are combined with the accelerometer and gyroscope data to efficiently track and classify gestures in a meaningful and intuitive way. The distance estimation of orientation data is more efficient and enables better discrimination among similar gestures patterns.

### 4.2. Hand Gesture Recognition Based on DTW

The inertial motion sensor input device is equipped with buttons for users to start and stop gestures. Users begin a gesture by pressing the button, and complete the gesture by releasing it. Each hand gesture training sample is collected in XML file format and stored in a template database for gesture recognition. We implement a multidimensional real-time gesture recognition algorithm using DTW. During the gesture training process, a template and threshold value for each class of gestures is computed. In the real-time recognition stage, the DTW algorithm measures the similarity between the input and the templates. The input can either be accepted as a member of the class to which it has the minimum normalized total warping distance, or if the similarity measurement does not match the threshold value, rejected as belonging to none of the classes.

A gesture template can be computed by recording a single or *N* training example(s) for each class of gestures that must be recognized. The template gesture for each class of gestures can be found from the recorded training examples by computing the distance between each of the *N* training examples. The training example in the given class, which has the minimum normalized total warping distance when compared against the *N*–1 training examples, is recognized as the template gesture for that class. The classification threshold value for each template gesture is calculated by taking the average total normalized warping distance between the template and the other *N* − 1 training examples for that gesture.

Using a classification threshold for each template gesture overcomes the problem of false positives during the recognition stage, as unknown time series input is classified as a null class if no match is found in the gesture database. If a new gesture class is added to an existing gesture in the database, or if an existing gesture is removed, the gesture model does not need to be retrained. Instead, we need only train a new template and threshold value for the new gesture, which thereby reduces the training time.

Once the DTW algorithm has been trained, an unknown multidimensional time-series input gesture can be classified by calculating the normalized total warping distance between the input and each of the gesture templates in the database. The input gesture is then classified according to the template class corresponding to the minimum normalized total warping distance.

The DTW process is described as follows. If X and Y are two time-series gesture sequences with different lengths, where $X=\left\{x_{1},x_{2},\ldots x_{n}\right\}\text{ and }Y=\left\{y_{1},y_{2},\ldots y_{m}\right\}$, a cumulative distance matrix D, which represents a mapping and alignment between X(i) and Y(j), is constructed to measure the similarity between sequences X and Y. Subsequently, a warping path $W=\left\{w_{1},w_{2},\ldots w_{P}\right\}$ comprised of the local cumulative distances D(i,j) is calculated. The length of the warping path is:

(15) max(n,m)≤P<n+m

and the kth element of the warping path is given by:

(16) $$w_{k}=(x_{i},y_{j})$$

To improve the efficiency of DTW, we constrain the warping path so that the maximum allowed warping path cannot drift too far from the diagonal. Controlling the size of the warping window speeds up the DTW computation. The constraints placed on the warping path are as follows. The warping path must start at the beginning of each time series, *i.e*., at $w_{1}=\left(1,1\right),$ and end at $w_{P}=(n,m)$. This ensures that every index of both time series is used in the warping path. The warping path must be continuous; *i.e*., if $w_{k}=(i,j)$, then $w_{k+1}$ must equal either (i,j), (i+1,j), (i,j+1), or (i+1,j+1). The warping path must exhibit monotonic behaviour; *i.e*., the warping path cannot move backwards. The optimal warping path that minimizes the normalized total warping distance is given by:

(17) $$d\left(W\right)=min\;\frac{1}{P}\overset{P}{\underset{k=1}{\sum }}d(w_{k_{i}},w_{k_{j}})$$

where $d(w_{k_{i}},w_{k_{j}})$ is the Euclidean distance between point i in time-series X and point j in time-series Y, is given by $w_{k}$. The minimum optimal total warping path can be effectively found using dynamic programming through the cumulative distance D(i,j) given by:

(18) $$D\left(i,j\right)=d\left(x_{i},\;y_{j}\right)+\text{min}\{D\left(i-1,j\right),D\left(i,j-1\right),D\left(i-1,j-1\right)\}$$

The DTW(X,Y) between the two time-series sequences is then calculated by finding the minimum normalized total warping distance between X and Y. This is defined as:

(19) $$DTW\left(X,Y\right)=\frac{1}{P}\overset{P}{\underset{k=1}{\sum }}d(w_{k_{i}},w_{k_{j}})$$

**Figure 6:**
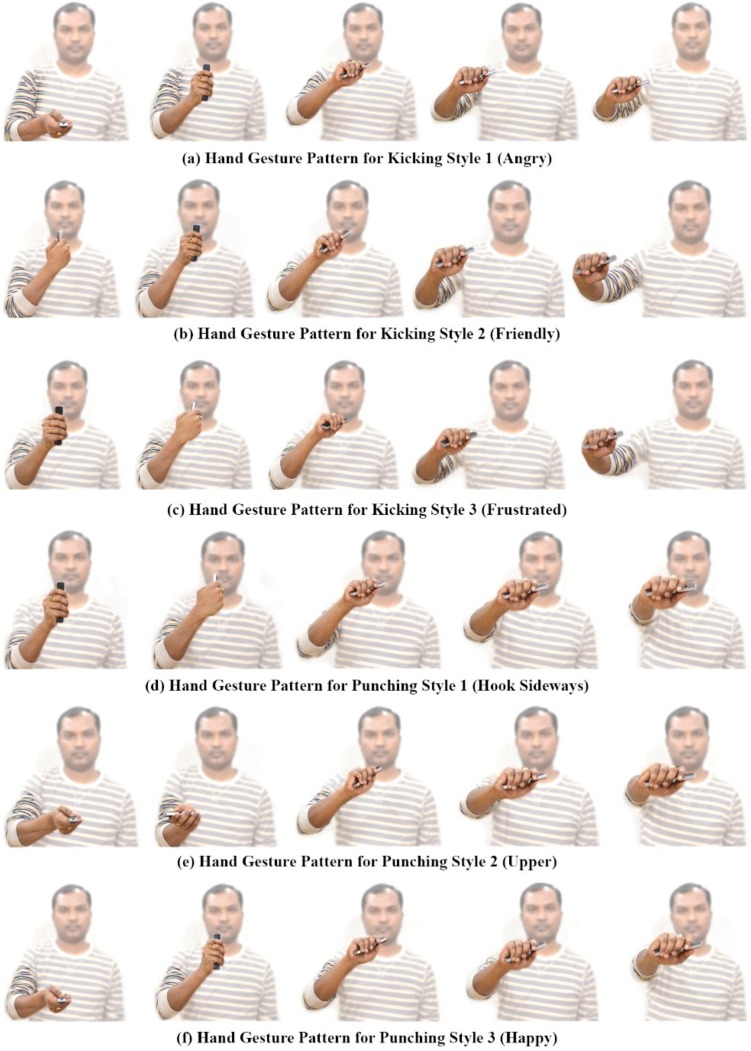
Mimic gesture patterns for kicking and punching actions.

Figure 6 shows the recorded freehand affordance mimic gesture patterns for different kicking and punching styles from the 6DOF wireless motion sensor. We use the term “mimic” to represent the animation action that the virtual avatar is going to perform. The gesture training templates used to mimic kicking and punching actions are generated as shown in Figure 6a–f. The user begins the gesture by pressing the start button on the wireless motion sensor, and then freely and continuously moves his/her hand in 3D space.

Users can make gesture segments to encode context and sub-context features of the gesture actions by pausing his/her hand motion for a fraction of a second, and then moving again until the gesture is completed. The completion of a gesture is signified by releasing the button on the wireless motion sensor. The proposed DTW-based recognition algorithm enables early recognition of gestures and estimates gesture variations of a live gesture according to the learned templates.

## 5. Expressive Motion Synthesis

Applications such as computer games and serious games use 3D gesture recognition for character animation, either to control avatars or interact with virtual worlds. In these applications, a small number of motion clips are used to play avatar animations. These clips are repeatedly played back whenever the instance of the given action is recognized through the interaction of user gestures. However, users often find that the animation is monotonous and unrealistic. Current animation systems lack the ability to recognize the user’s intentions and interactively produce an appropriate reaction by the system.

**Figure 7:**
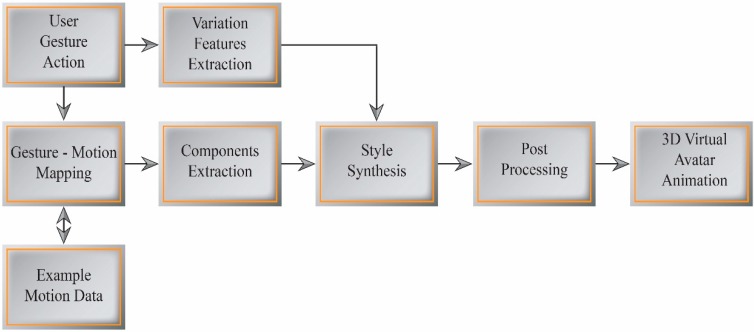
Expressive motion synthesis.

Motion graph and motion synthesis techniques [33] typically synthesize actions by combining motion segments from a database, or adjust motion through statistical modeling and cannot synthesize variations in motion. However, users sometimes require a motion with a style and variation that is not in the database. We aim to produce a dynamic and interactive interface technique for authoring and controlling avatar motion. Our expressive motion synthesis approach facilitates the interactive control of virtual avatar behaviors in virtual worlds. Figure 7 illustrates our expressive motion synthesis process using the dynamic gesture mapping interface.

### 5.1. Gesture-Motion Mapping

Human motion is generally continuous and smooth. For proper character animation, the bones and joints must follow a logical hierarchy. Each joint has one or more DOFs that define its possible range of motion. Specifying values for these DOFs results in a particular body pose; changing these values over time results in movement. Human motion data comprise a high-dimensional time series. The hierarchy structure of each frame can be represented as root positions and joint orientations. Motion is defined as a continuous function of frame indexes to poses of the avatar skeleton. This can be written as:

(20) $$m\left(t\right)=\{p\left(t\right),q_{1}\left(t\right),\ldots ,q_{n}\left(t\right)\}$$

where t is the frame index, p denotes the position of the root joint, and $q_{n}$ is the joint orientation.

Our gesture-based interactive avatar control application system uses a 3D human model from the Rocketbox Libraries with a hierarchical structure for character animation. We used 18 important joints from the avatar skeleton to control expressive movement. A small database of example movements for this system was obtained from conventional key-frame techniques and freely available motion capture databases, such as the Carnegie Mellon University (CMU) motion capture database. The motion data are resampled and simplified to the skeleton structure of a 3D virtual avatar.

**Figure 8:**
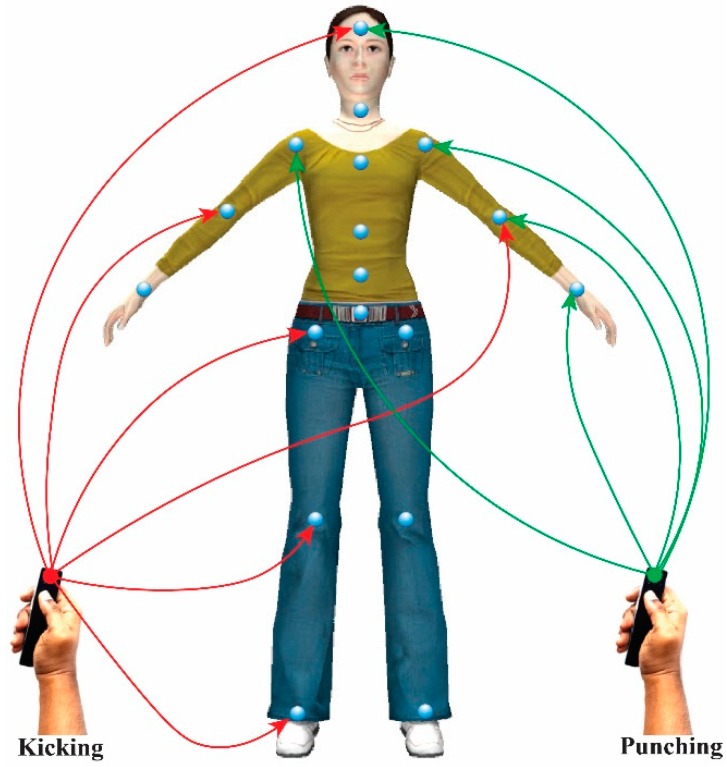
Avatar control using dynamic gesture mapping.

Our system supports both individual and group control of avatar joints using the inertial motion sensor in real time, like an avatar puppetry system [34]. However, our dynamic gesture mapping interface enables users to seamlessly control the avatar motion with their hand gestures. The avatar joints are controlled in groups based on the skeleton hierarchy, such as right leg, left leg, right arm, left arm, and torso-head, or individually. This reduces the complexity of motion synthesis, and simplifies the interaction. This system gives users the freedom to control the avatar joints of their choice, depending on the context of motion.

Our system tracks user hand gestures. A dynamic interface maps the gestures to the corresponding joint parameters of the 3D virtual avatar to control and synthesize a new style of expressive avatar motion. The gesture-motion mapping technique employs an embodied interaction with the mimic gesture patterns for each type of avatar action. The mimic gesture action templates are embedded in significant and specific joint parameters of the avatar body, *i.e*., those that most effectively convey each desired motion, and provide control over expressive characteristics of avatar movement depending on the context and sub-context of the gesture. These joints are encoded as candidate joints for the control of avatar motion in real time using hand gestures, as shown in Figure 8. User hand gestures are transferred to the avatar joint parameters by mapping the extracted features from the inertial motion sensor data.

### 5.2. Components and Features Extraction

Style can be regarded as a subtle variation of basic motion. The style and content of a motion are independent and can be separated. We assume that the motion data are generated from a few dimensional feature vectors, and that these features are statistically independent. The motion data can be represented as a time-series vector or a set of samples of random variables. By decomposing the joint angle data of full-body motion that has been fitted to a hierarchical skeleton, we parameterize the motion into independent joint components. Similarly, the hand gesture data are decomposed to obtain dynamic gesture components.

We use ICA to extract the motion components from significant candidate joints and decompose user hand gestures into independent components. PCA is used as a form of preprocessing to determine the dimensionality of the motion features. This simplifies the gesture-to-motion mapping procedure, and reduces the computational complexity. We used Euler angles, representing the rotation of the candidate joints provided in the motion capture data, as well as orientation data from the hand gestures in the form of Euler angles, for ICA decomposition to obtain corresponding motion components.

The process of extracting independent components using ICA can be described as follows. Given input motion data m(t), we apply ICA to compute the independent component c(t) and the corresponding mixing matrix A as:

(21) m(t)=Ac(t)

We employ FastICA [35] algorithm to decompose the motion data into independent components. Before applying the ICA algorithm, the motion data undergoes two preprocessing steps. First, the data are centered around their statistical mean. Then, the centered data are whitened using PCA. Whitening linearly transforms the data into a set of uncorrelated components. The number of principle components determines the number of independent components. Related details are provided in [36]. The complete ICA model can be expressed as:

(22) m(t)=E{m}+PAc(t)

where E{m} is the mean of the input data and P is the PCA matrix used for whitening.

The dynamic gesture mapping interface selects an example motion from the motion database by classifying each input gesture. To extract motion components from high-dimensional example motion data, we use the encoded information of each gesture action, such as context, sub-context, and candidate joints. The DTW-based gesture recognition algorithm classifies the performed gesture, and extracts variation features from the recognized reference. The real-time estimation of dynamic gesture variations at a given instant enables the real-time expressive modulation of multiple joint parameters. The extracted variation features represent changes in speed, duration, and orientation. These gesture variations are used to expressively control the avatar motion in real time.

### 5.3. Style Synthesis

ICA is applied to the candidate joints independently specified by the mapping relationship between the user gesture action and the example motion to extract motion components. The extracted significant joint motion components are combined with extracted dynamic gesture components from the hand gesture data to synthesize new, realistic avatar motions in the ICA domain. Our system extracts independent components from each body part specified by the user gesture-motion mapping, which provides users with fine control of mixing components from the hand gesture motion and produces a rich variety of styles and variations for each body part.

Several mathematical operations can be used to edit motion components by using gesture components to generate a new style of avatar motion sequences. The motion editing operations used in our system enable users to control the candidate joints for specific key frames, or for continuous control over a period of time. The user-specified dynamic gesture components from hand gestures are mapped to the joint motion components of the example motion, such that fine details are preserved and blended over time to achieve a new style of motion. This editing operation is mathematically expressed as:

(23) $$m^{\prime }\left(t\right)=E\left\{m\right\}+{\left(PA\right)}_{1}(c\left(t\right)-c_{1}\left(t\right))+\alpha {\left(PA\right)}_{1}c_{1}\left(t\right)+\left(1-\alpha \right){\left(PA\right)}_{2}c_{2}\left(t\right)$$

where m′(t) is the edited motion, E{m} is the mean of the input motion data, c(t) is its independent component, and $c_{1}\left(t\right)$ is the selected joint independent component with mixing matrix ${\left(PA\right)}_{1}$. $c_{2}\left(t\right)$ is the independent component of the hand gesture motion and ${\left(PA\right)}_{2}$ is its mixing matrix. α is a blending parameter for controlling the editing process. Similarly, other editing operations such as adding, tuning, and transferring components can be used to obtain interesting results depending on the motion requirements.

After manipulation, the motion data is post-processed to correct statistical artifacts in the edited motion by preserving the joint angles from the original data; avatar motion is reconstructed by adding the motion data removed prior to motion decomposition. We impose a predefined orientation limit and DOF for each joint to prevent unnatural joint motions. The synthesis of styles in the ICA domain has several limitations. This method is more effective for cyclic motions than acyclic motions, because it is easier to align cyclic motions than arbitrary ones. However, if we properly perform the decomposition to obtain cyclic aspects from arbitrary motions, we can produce effective results.

## 6. Experimental Results

Our interactive avatar control application uses the dynamic gesture interface system. Interaction with the application involves performing a gesture to generate a specific style of avatar motion. It additionally involves the continuous manipulation of that stylistic avatar motion by extracting meaningful variations from the gesture execution. Gesture actions are similar but not exact; variations are primarily due to differences among individuals. Our dynamic gesture interface extracts the intention of a gesture, and generates the user-desired results in avatar motions with fine control of avatar joint parameters.

We demonstrated our dynamic gesture-based interactive control interface system using the motion example of kicking and punching with the mimic gesture patterns. Our system software was programmed in C#, and uses the Unity3D game engine to render the 3D virtual avatar. The system was run on a PC with 16 GB of memory and an Intel Core i7 with a 3.40 GHz CPU.

### 6.1. Style Variations in Avatar Motion

**Figure 9:**
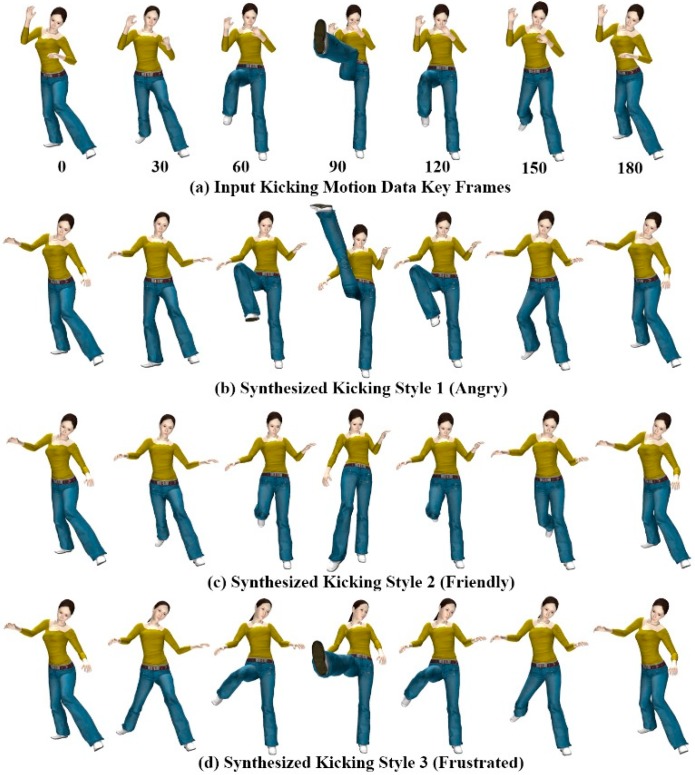
Kicking motions of avatar.

Figure 9 shows three styles of kicking motion generated using the gesture patterns provided for each style of motion. All three motion styles Figure 9b–d were generated using a single example motion (Figure 9a), and mimic hand gesture patterns in Figure 6a–c for the kicking motions. For kicking style 1 (angry) and style 2 (friendly), we selected the right leg part and both the right and left forearm joints as candidate joints for extracting corresponding motion components from the input motion data. For kicking style 3 (frustrated), we selected the head in addition to the style 1 and 2 candidate joints.

**Figure 10:**
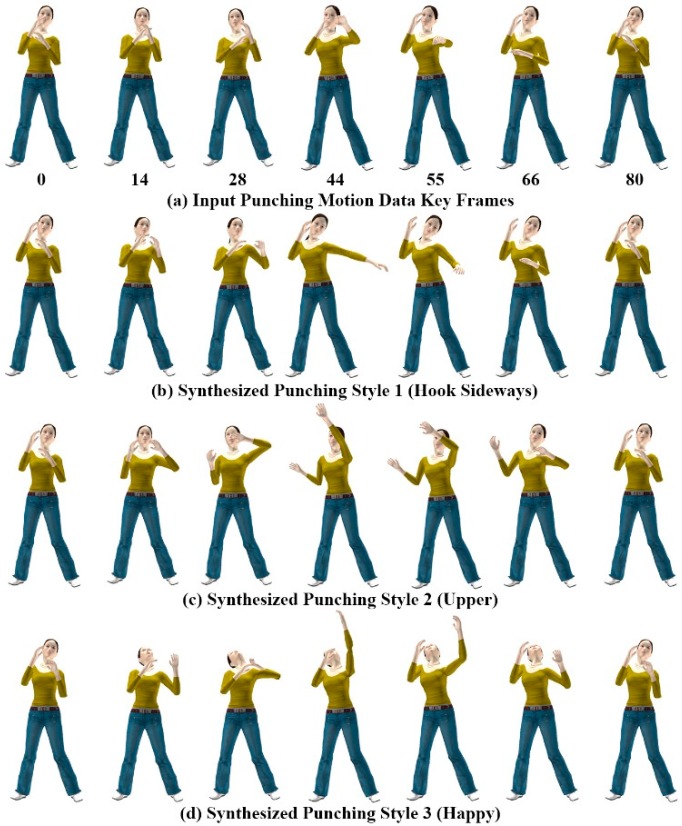
Punching motions of avatar.

A rich set of new motion styles can be synthesized depending on user gesture-motion mapping relationships. The avatar’s motion trajectory changes according to the user’s gesture-motion mapping relationship, which alters the style of the motion. Figure 10 shows different styles of the punching motion generated using the gesture patterns provided for each style of motion. All three motion styles in Figure 10b–d were generated using the single example motion in Figure 10a, and mimic the hand gesture patterns of Figure 6d–f for the punching motions.

**Figure 11:**
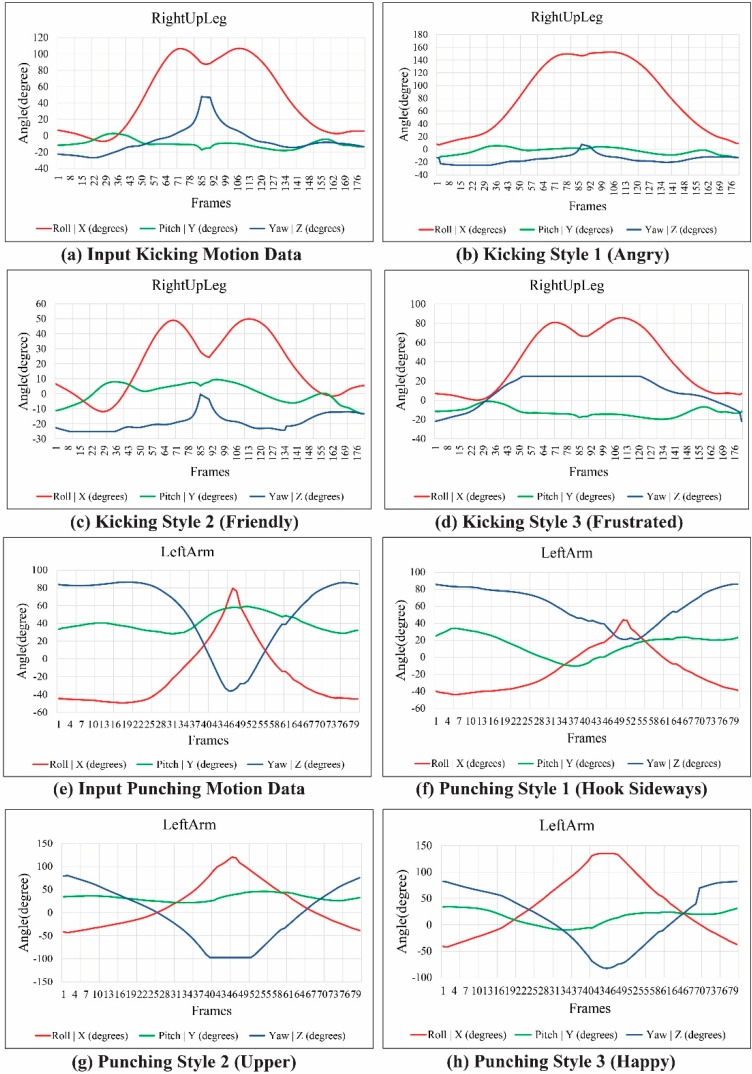
Motion curves of RightUpLeg and LeftArm joints.

Figure 11 shows the modulated and reconstructed motion curves of the RightUpLeg and LeftArm joints for each kicking and punching style of motion, synthesized from the example motion. The style of motion was modulated by deforming the joint motion trajectories with hand gesture data. From a single example motion, we created an adequate variety of interesting motions in the avatar using a combination of ICA-based analysis and DTW-based gesture recognition for gesture-motion mapping.

### 6.2. Spatial and Temporal Variations in Motion

**Figure 12:**
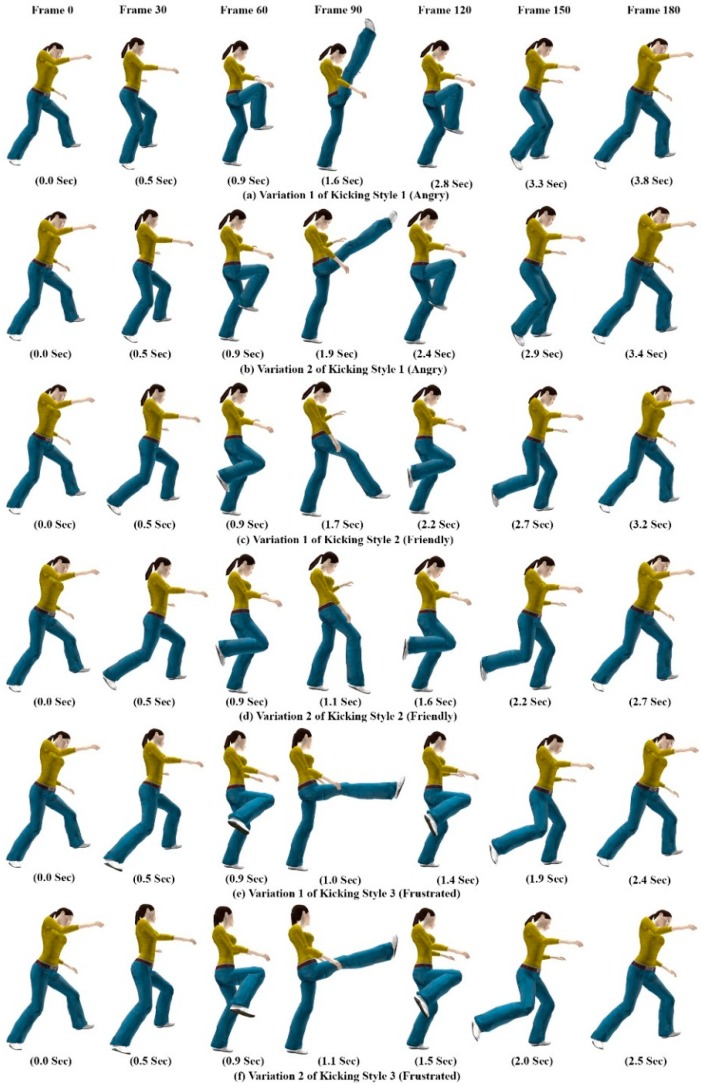
Spatial and temporal variations in kicking motions of avatar.

The proposed system produces rich and continuous variations for each style of avatar motion in time and space. Hence, our system increases the reusability and flexibility of motion data. Similar gesture patterns can generate an unlimited number of motion variations depending on user-supplied components and variation features provided through gesture execution. Figure 12 shows the spatial-temporal variations of the kicking motion obtained for each style by mapping hand gesture variation features to avatar motion parameters. These parameters were then continuously modulated depending on how the gesture was performed.

The inter-class gestures show how we perceived each style of motion; the intra-class variability demonstrates our dynamic way of producing the same motion. Users can make new styles and variations in avatar motion by selecting a new group of joints with new gesture patterns. They can then create a new combination of gestures and a new motion class. Thus, our system enables users to introduce new motions to meet their specific requirements.

### 6.3. Evaluation and User Study

We performed a gesture recognition experiment to test and evaluate the efficiency of the inertial motion sensor for the hand gesture patterns shown in Figure 6. Table 1 presents the confusion matrix table for the target gestures. Columns represent recognized gestures, and rows denote the actual input gestures. An average accurate recognition rate of 97.6% was achieved using the DTW algorithm. The combination of acceleration and orientation data as feature parameters, with segmentation of the gesture action into candidate gesture templates for gesture recognition, enables users to produce affordance gesture input.

**Table 1:** Confusion matrix of target gestures.

	Gesture 1	Gesture 2	Gesture 3	Gesture 4	Gesture 5	Gesture 6
**Gesture 1**	**0.97**	0	0	0	0	0.03
**Gesture 2**	0	**0.99**	0	0	0.01	0
**Gesture 3**	0	0	**0.97**	0.03	0	0
**Gesture 4**	0	0	0.02	**0.98**	0	0
**Gesture 5**	0.01	0	0	0	**0.99**	0
**Gesture 6**	0.04	0	0	0	0	**0.96**

The system was tested by several users who had minimal or no experience with 3D animation. We asked participants to test the system by providing instructions for mimic hand gesture actions for a kicking motion to generate stylistic kicking motions for an avatar. The users successfully produced the stylistic kicking motions of the avatar at interactive speeds in approximately 4–5 min.

The results show that our dynamic gesture interface provides continuous and rich interaction. The gesture-based interaction technique gives the sense of engagement and playful behavior for controlling avatar motion. Synthesis of expressive avatar motions can be spontaneously generated and varied. This enables even novice users to quickly and easily control and synthesize realistic avatar animation at interactive speeds. The generated avatar motions are realistic and perceptually valid; moreover, they can be effectively conveyed and expressed in interactive applications, such as virtual worlds, computer games, humanoid interfaces, and other virtual environments.

## 7. Conclusions

In this paper, we presented a dynamic gesture-based interactive interface system for authoring and controlling the motion of a 3D virtual avatar using a single inertial motion sensor. The proposed touch and shake metaphor extracts meaningful information from user hand motions. A DTW-based gesture-motion mapping interface, and the expressive synthesis of new stylistic motions by mapping dynamic gestures using an ICA-based decomposition method, enables users to change the style and behavior of avatar motions from a single example motion, which increases the reusability and flexibility of the motion database. The real-time estimation of dynamic gesture variations enables users to spontaneously and expressively control avatar motion with variations. This method is suitable for interactive applications, such as computer games and non-verbal communication via virtual avatars. The system’s combination of gesture recognition with gesture variation tracking allows effective control and continuous interaction with a virtual environment.

In future work, we will further develop and enhance our interface method for authoring and controlling an avatar and its motion. Our future work will also enhance the variety of personalized hand gestures and improve the recognition rate. Further, we plan to test the intuitiveness and naturalness of the system by incorporating wide variety of hand gestures with more avatar example motions through usability evaluations. Exploring different ways of tracking user hand motions, and combining our interface with other hand motion sensing devices, is one possible approach for future work. The proposed gesture-based interaction technique could also be examined in other natural interactive applications such as sign language and hand writing recognition.
